# Barriers and facilitators to weight loss in patients living with obesity and OSAHS: a qualitative study based on the Theoretical Domains Framework (TDF)

**DOI:** 10.3389/fmed.2026.1782833

**Published:** 2026-04-24

**Authors:** Yi Wen, Shuanglan Lin, Xia Yang, Shiqi Xie, Jianrong Zhou, Jinglan Chen

**Affiliations:** 1 School of Nursing, Chongqing Medical University, Chongqing, China; 2 School of Social Development and Public Policy, Fudan University, Shanghai, China; 3 Department of Eye and Otorhinolaryngology, Women and Children’s Hospital of Chongqing Medical University, Chongqing, China

**Keywords:** obesity, obstructive sleep apnea-hypopnea syndrome, qualitative study, theoretical domains framework, weight loss

## Abstract

**Background:**

Weight loss represents a cornerstone therapy for individuals living with obesity and obstructive sleep apnea-hypopnea syndrome (OSAHS). However, long-term effectiveness is often limited by complex behavioral barriers, with over 70% of patients experiencing weight regain. This study explored patient-perceived barriers to sustainable weight loss and modifiable facilitators to clarify the interplay among cognitive, behavioral, and environmental factors in this population.

**Methods:**

This qualitative study employed the Theoretical Domains Framework (TDF) guided by a critical realist epistemology. Semi-structured interviews were conducted with 26 purposively recruited participants living with obesity and OSAHS (mean age 43.2 ± 9.9 years, 73.1% male) from a tertiary sleep clinic in China. Recruitment continued until information power was achieved. Data were analyzed using abductive thematic analysis following Braun and Clarke’s six-phase framework, with coding moving iteratively between deductive TDF domain assignment and inductive theme development.

**Results:**

Analysis mapped barriers and facilitators onto all 14 TDF domains. Key findings included knowledge misconceptions that actively shaped treatment preferences, a lack of behavioral repertoire rather than motivation driving unsuccessful attempts, and passive drift from goals indicating self-regulatory deficits. Environmental disruptions evoked helplessness, while social influences showed dual effects: supportive family enhanced adherence, yet well-intentioned behaviors sometimes undermined efforts. Facilitators included personal experience-grounded knowledge, health-related anxiety as a motivator, and desire for structured professional guidance.

**Conclusion:**

This first application of the TDF to OSAHS weight management identified key intervention targets across capability, opportunity, and motivation dimensions. The findings provide preliminary evidence for developing theory-informed, multidimensional strategies and support a shift toward structured, personalized interventions. Given the exploratory nature of this study, these conclusions warrant further investigation in diverse populations.

## 1 Introduction

Obstructive sleep apnea-hypopnea syndrome (OSAHS) is a prevalent sleep-related respiratory disorder characterized by snoring, excessive daytime sleepiness (EDS), fatigue, and other clinical symptoms (1, 2). Recent systematic review indicates that OSAHS affects approximately 9%–38% of the general adult population (3). This condition is associated with significant comorbidities, including cognitive impairment, stroke, and various cardiovascular disorders such as heart failure, arrhythmia, and coronary artery disease (4–6). Furthermore, OSAHS patients demonstrate an elevated risk of motor vehicle accidents (7, 8). The pathogenesis of OSAHS involves multiple risk factors, including age, gender, excess body weight, race, and craniofacial anatomy, with obesity being the most well-established and significant contributor (9–11). The Wisconsin Sleep Cohort study demonstrated that a 10% weight gain corresponds to an approximate 32% increase in the apnea-hypopnea index (AHI), resulting in a sixfold greater likelihood of developing moderate-to-severe OSAHS (12). The frequent co-occurrence of OSAHS and obesity exerts a synergistic effect that substantially exacerbates cardiovascular and metabolic complications beyond the impact of either condition alone (13). Notably, obesity represents a modifiable risk factor for OSAHS (14). Meta-analytic evidence confirms that weight reduction significantly improves key OSAHS parameters, including AHI, oxygen desaturation index (ODI), and EDS, while enhancing overall patient health (15–17). In recognition of these findings and considering the prevalent adverse lifestyle patterns among patients, weight loss has been recognized as a primary therapeutic intervention for individuals living with both obesity and OSAHS.

Despite compelling evidence demonstrating that weight loss effectively reduces the AHI and confers additional health benefits, behavioral modification-based weight loss interventions consistently yield suboptimal success rates, with weight regain representing a common clinical challenge (18, 19). This phenomenon of weight loss failure predominantly stems from limitations in intervention program accessibility and implementation (20). The complex process of weight management in individuals living with obesity and OSAHS involves a dynamic interaction among multiple determinants across cognitive (e.g., knowledge, beliefs), psychological (e.g., motivation, self-efficacy), social (e.g., support systems), and environmental (e.g., resource availability) domains. Understanding these multidimensional influences is essential for developing effective interventions. However, the specific symptoms of OSAHS, such as excessive daytime sleepiness and fatigue, may directly impair patients’ energy levels and self-regulatory capacity, thereby introducing disease-specific barriers that extend beyond those typically encountered in general obesity management.

The Theoretical Domains Framework (TDF) represents a systematic approach for integrating behavioral change theories, originally developed by Michie and colleagues in 2011 to provide comprehensive theoretical foundations for health behavior intervention research (21). This framework synthesizes essential constructs derived from 33 established psychological and behavioral change theories, ultimately consolidating them into 14 core theoretical domains (22). These domains comprehensively address the cognitive, emotional, social, and environmental dimensions that collectively influence individual health behavior modification, thereby offering researchers a structured analytical methodology for systematically identifying both barriers and facilitators to behavioral change. Unlike traditional single-theory approaches that typically capture only selected dimensions of behavior, the TDF’s integrative design enables a comprehensive examination of the multiple determinants influencing health-related actions. This broad analytical scope is particularly valuable for understanding complex behaviors in chronic disease management, where cognitive, affective, social, and environmental factors interact in dynamic ways (22). Within chronic disease management research, the TDF has established substantial empirical validity. Illustrative applications include its use in gestational health management studies to delineate critical barriers impeding healthy dietary habits and physical activity among pregnant women (23), as well as in post-gestational diabetes research to clarify fundamental factors affecting postpartum weight management (24). These demonstrated applications collectively substantiate the framework’s utility and efficacy in health behavior investigation.

For the specific context of weight management in OSAHS, the TDF offers distinct advantages. The framework’s 14 domains encompass both generic behavioral determinants and constructs particularly relevant to this population. Its capacity to systematically capture how OSAHS symptoms such as fatigue and daytime sleepiness affect patients’ capacity for behavioral change makes it especially well-suited for disentangling the complex interactions in this population. Notably, while previous research has applied the TDF to various chronic conditions, no studies have systematically employed this framework to investigate weight loss determinants in patients with OSAHS. This gap is significant because the framework’s comprehensive scope aligns with the multifactorial nature of weight management challenges in this population, where disease symptoms, psychological factors, and environmental influences converge.

Building upon this theoretical foundation, the present study employs the Theoretical Domains Framework (TDF) as an analytical tool to systematically investigate the key determinants influencing weight reduction in patients living with obesity and OSAHS. The research aimed to identify patient-perceived barriers to sustainable weight loss and modifiable facilitators of treatment adherence, thereby elucidating the complex interactions among cognitive, behavioral, and environmental factors in weight management for this specific population.

## 2 Materials and methods

### 2.1 Study design

This study employed semi-structured interviews rooted in a critical realist epistemology and a subtle realist ontology, acknowledging that while a reality exists regarding patients’ weight loss experiences, our access to it is mediated by participants’ interpretations and our analytical lens. The investigation was guided by the Theoretical Domains Framework (TDF) to systematically explore weight loss determinants among individuals living with obesity and OSAHS. The TDF served as the guiding framework for both data collection and analysis, providing a scaffold to identify and map barriers and facilitators. The interview guide was developed by operationalizing each TDF domain into open-ended questions designed to elicit participants’ experiences. The initial draft was reviewed by the research team for content and face validity. The interview protocol (Supplementary Appendix 1) underwent pilot testing with two individuals living with obesity and OSAHS to validate question clarity and content appropriateness. During pilot testing, we assessed both question comprehensibility and whether questions effectively elicited responses relevant to the intended TDF domains. Based on participant feedback and interviewer observations, we identified and refined questions that were too abstract or elicited redundant information, enhancing comprehensibility while maintaining theoretical fidelity. To ensure methodological rigor and transparency, the entire research process from study design to data analysis strictly adhered to the Consolidated Criteria for Reporting Qualitative Research (COREQ) guidelines (25), with complete documentation provided in Supplementary Appendix 2. This approach ensured theoretical grounding and epistemological coherence. The TDF served as the guiding framework for both data collection and analysis, providing a scaffold to identify and map barriers and facilitators.

### 2.2 Participants

Employing a purposive sampling strategy, we recruited eligible participants from the sleep laboratory at the Department of Otorhinolaryngology in a tertiary hospital in Chongqing, China. Purposive sampling was chosen to ensure the inclusion of participants with diverse experiences and perspectives relevant to the research aim. The inclusion criteria are as follows: (1) diagnosed with OSAHS (AHI ≥ 5 events/h); (2) meeting the Chinese obesity criteria (body mass index, BMI) ≥ 28 kg/m^2^ (26), equivalent to WHO Class I obesity (BMI ≥ 30 kg/m^2^) (27); (3) individuals aged 18–60 years; (4) able to give informed consent. Participants with cognitive impairments and unable to communicate were excluded. We employed a purposive sampling strategy to recruit participants with maximum variation in experiences pertinent to weight loss challenges, ensuring a comprehensive exploration of barriers and facilitators. A trained research nurse approached consecutive eligible patients during routine sleep laboratory visits. Study purpose and procedures were explained, and written information sheets were provided. Of 29 patients approached, 26 consented (89.7% participation rate). Three declined due to time constraints or lack of interest. No financial incentives were offered.

### 2.3 Interview procedures

The research team comprising three trained investigators conducted all face-to-face interviews in a designated quiet room at the clinical facility between March and June 2022, with each session audio-recorded to ensure data integrity. Interviewers held postgraduate qualifications and had received specialized training in qualitative research methodologies, though no prior relationships existed with participants to maintain objectivity. During the interviews, researchers employed probing techniques to elicit comprehensive responses while contemporaneously documenting observational field notes. Following each interview, participants completed a standardized demographic and clinical profile questionnaire to provide rich contextual data, enabling readers to assess transferability. Recruitment continued until information power was achieved, as conceptualized by Malterud et al. (28). We determined that our study’s narrow aim, specific sample, use of the established TDF framework, and high-quality dialogue provided strong information power. To operationalize this, the research team convened after every 3–4 interviews to review the data. Recruitment ceased when two consecutive review meetings concluded that no new TDF-coded subthemes were emerging, and further interviews were adding minimal novel insights relevant to the research question. Data collection ceased when the research team consensus indicated that the forthcoming interviews were adding minimal novel insights relevant to the TDF domains. Individual interviews averaged approximately 40 min in duration, with each participant contributing to a single interview session to maintain data consistency across the sample.

### 2.4 Ethical considerations

The study protocol received ethical approval from the Institutional Review Board of the First Affiliated Hospital of Chongqing Medical University (Refer No. 2022-052). Prior to participation, all subjects were fully briefed regarding the study objectives, significance, and confidentiality measures. Written informed consent was obtained from each participant, specifically authorizing audio recording and use of anonymized transcript excerpts. Participants retained the unconditional right to withdraw from the study at any point during the interview process without consequence.

### 2.5 Data analysis

The data were analyzed using thematic analysis following the steps outlined by Braun and Clarke (29, 30). This method was chosen for its systematic and theoretically flexible approach to identifying, analyzing, and reporting patterns within qualitative data, making it well-suited for theory-informed research. Consistent with our critical realist stance, we employed both semantic and latent coding. Semantic coding allowed us to stay close to participants’ explicit statements, capturing surface-level meanings and experiences. Latent coding enabled us to interpret underlying ideas, assumptions, and patterns, thereby generating deeper interpretive insights. This dual approach was essential for moving beyond description to reveal the mechanisms through which barriers and facilitators operate within the context of OSAHS.

**Phase 1: Familiarizing with the data**. All interviews were transcribed verbatim within 24 h by a trained researcher. The transcripts were repeatedly checked against audio recordings for accuracy by the research team but were not returned to participants for verification. The research team immersed themselves in the data by reading and re-reading the transcripts and recording initial ideas.

**Phase 2: Generating initial codes**. We conducted a theoretically-grounded thematic analysis. We adopted an abductive approach, meaning our coding process moved iteratively between the data and the 14 TDF domains. We began with deductive coding, using the TDF domains as a pre-defined framework to categorize and tag the data. Concurrently, we remained open to patterns within the data that could refine or elaborate the application of the TDF domains, engaging in inductive coding. This phase was conducted using NVivo 12.0 software, generating a large set of initial codes.

**Phase 3: Searching for Themes**. The aim of this phase was to collate the initial codes into potential themes and subthemes. The research team engaged in deep discussion of the coded data, comparing codes across different data extracts and searching for recurring patterns, relationships, and conceptual links. We focused specifically on clusters of codes that spoke to the core research question regarding factors influencing weight loss in patients with OSAHS. Through this process, we constructed preliminary maps of themes and subthemes within each TDF domain.

**Phase 4: Reviewing Themes**. We reviewed the candidate themes at two levels. First, at the level of the coded data extracts, we checked if the extracts formed a coherent and meaningful pattern under each candidate theme. Second, at the level of the entire dataset, we reviewed the thematic map to assess whether it accurately reflected the meanings evident in the data set and whether the themes were distinct and meaningful. During this phase, some themes were merged, split, or discarded.

**Phase 5: Defining and Naming Themes**. We developed a detailed analytical narrative for each identified theme and subtheme, precisely defining the core idea of each and its relation to the research question. Themes were given concise and informative names that immediately conveyed their essence. For example, within the “Knowledge” domain, a barrier subtheme was definitively named “Limited understanding of OSAHS and its relationship to obesity, coupled with the misconception that surgery is the only effective treatment.”

**Phase 6: Producing the Report**. The final analytic output was a set of themes and subthemes organized under the relevant TDF domains. This structure guided the presentation of findings in the Section “3 Results” (see Table 1 for a comprehensive mapping). Throughout the process, coding disagreements were resolved through consensus discussions. Persistent disagreements were adjudicated by a senior researcher (S.X. or J.Z.). An audit trail of all decisions was maintained.

**TABLE 1:** Mapping of subthemes to TDF domains.

TDF domains	Subthemes (barrier)	Subthemes (facilitator)
Knowledge	Limited understanding of OSAHS and its relationship to obesity	Accurate knowledge as a foundation for motivation
Skills/behavioral regulation	Unfamiliarity with effective weight management techniques	None
Beliefs about consequences	None	Positive outcome expectancies driving behavior Dual motivational pathways Multifaceted nature of outcome expectations
Social/professional role and identity	None	Self-regulation as a core identity commitment
Intentions	Structural barriers undermining intention formation Underestimation of disease severity as a barrier to intention formation	Strong, health-anchored intentions
Reinforcement	Intercurrent health problems as negative reinforcers	External reinforcement through social support Internal reinforcement through habit formation
Beliefs about capabilities/optimism	None	Self-efficacy derived from past successes
Memory, attention, and decision process	Forgetting and the failure of self-monitoring	None
Environmental context and resources	Pervasive environmental disruption of weight loss efforts	None
Social influences	Ambivalent or counterproductive social support	Desire for supportive accountability
Emotions	Emotional states as barriers to adherence Weight loss distress and burn-out Emotional detachment as a protective mechanism	Health-related anxiety as a motivator
Goals	None	Specific goals with flexible implementation

TDF, Theoretical Domains Framework; OSAHS, obstructive sleep apnea-hypopnea syndrome.

### 2.6 Quality and rigor

The study employed Guba and Lincoln’s framework (31) to ensure methodological rigor through four established criteria. Credibility was achieved through verbatim interview transcripts and detailed field notes, systematic iterative analysis, and maintenance of a detailed audit trail documenting all analytical decisions. Transferability was enhanced by comprehensive documentation of participant characteristics, sampling methodology, data collection, and analysis procedures. Dependability was strengthened through external protocol review by an independent researcher and verification of findings by investigators not involved in data collection. Confirmability was maintained through systematic recording of reflexive reports throughout the research process and by clearly delineating how the TDF shaped but did not dictate the findings.

## 3 Results

### 3.1 Participants

All eligible participants approached in the sleep laboratory consented to participate, with information power achieved following 26 interviews. The sample comprised 19 males (73.1%) and 7 females (26.9%), with a mean age of 43 years (range: 29–58). Complete demographic and clinical characteristics are presented in Table 2.

**TABLE 2:** Sociodemographic characteristics of participants (*n* = 26).

Variables	Mean ± SD or *n* (%)
Gender	
Male	19 (73.1)
Female	7 (26.9)
Age (years)	43.2 ± 9.9
BMI (kg/m^2^)	30.7 ± 2.8
AHI (events/h)	47.7 ± 23.3
OSAHS severity	
Mild	4 (15.4)
Moderate	1 (3.8)
Severe	21 (80.8)
Numbers of comorbidities	
0∼1	17 (65.4)
2∼4	9 (34.6)

BMI, body mass index; AHI, apnea-hypopnea index; OSAHS, obstructive sleep apnea-hypopnea syndrome.

### 3.2 Identified TDF domains and thematic structure

Our analysis mapped patient-reported experiences onto all 14 domains of the TDF, revealing a complex interplay of factors. Barriers were identified across eight domains, primarily relating to Capability (e.g., Knowledge, Skills) and Opportunity (e.g., Environmental context, Social influences). Facilitators spanned nine domains, with strong representation in the Motivation component (e.g., Beliefs about consequences, Intentions, Emotions). This mapping indicates that while motivation is crucial, deficits in practical capability and unsupportive environments frequently undermine weight loss efforts. Table 1 provides a comprehensive overview. The following sections present an interpretive analysis of these themes, elucidating not just what factors are present, but how they operate within the specific context of OSAHS. Below we present detailed interpretive analysis organized by domain.

### 3.3 Barriers

#### 3.3.1 Knowledge

Limited understanding of OSAHS and its relationship to obesity: several participants demonstrated significant knowledge gaps regarding OSAHS, its health consequences, and its association with obesity. This lack of awareness undermined motivation as they perceived no direct link between weight management and disease improvement. Without a clear cognitive connection between diagnosis and modifiable behavior, lifestyle modification was not prioritized.

From Knowledge Gaps to Active Misconceptions: The Belief in Surgery as the Sole Solution. Our analysis revealed that knowledge deficits were not merely passive gaps. In the absence of accurate information, specific knowledge gaps (e.g., about the efficacy of behavioral weight loss) can solidify into active misconceptions, which in turn directly shape treatment preferences that conflict with evidence-based recommendations. A small subset held a particularly consequential misconception that surgery was the sole effective treatment. This represents a belief system that may compete with medical advice, thereby devaluing behavioral (weight loss) management–a cornerstone of OSAHS therapy. The “silent” nature of early OSAHS, where palpable symptoms are absent from the patient’s perception, may make a “quick fix” like surgery appear more appealing than long-term lifestyle change.

*“I don’t know much about this disease (OSAHS), and I think there is no relationship between the two (obesity and OSAHS). I think obesity may be mainly related to blood pressure.”* P11

“I think that just losing weight may not be able to achieve a good therapeutic effect, you know, I think it may be necessary to do surgery.” P17

#### 3.3.2 Skills/behavioral regulation

Lack of effective weight management skills and self-regulation strategies: Among participants with prior weight loss attempts, a prominent theme was the absence of structured, evidence-based skills. Their efforts were characterized by *ad hoc*, overly restrictive attempts that proved unsustainable, leading to cycles of weight loss and regain accompanied by frustration and helplessness. Critically, these participants did not lack motivation. Rather, they lacked the specific behavioral repertoire and self-regulation strategies necessary for sustained weight management. Their expressed desire for systematic professional guidance underscores the need for interventions that build practical, individualized, and sustainable skills, not just provide information.

*“I used to eat less at noon and try not to eat meat at night. It lasted about a month, and the weight decreased a little. But it’s too painful not to let me eat meat. I couldn’t stick, so I got fat again.”* P8

#### 3.3.3 Intentions

While some participants expressed a general willingness to lose weight, this intention was often fragile and easily overridden by competing demands. Occupational constraints such as sedentary jobs or irregular hours were described as insurmountable obstacles. For these individuals, the perceived structural impossibility of action precluded the formation of strong behavioral intention. This finding points to a conceptual distinction between forming an intention and sustaining it when structural barriers are present. Interventions focused solely on strengthening motivation may fail if they do not simultaneously equip patients with strategies to navigate environmental constraints.

*“I want to lose weight, but my job is driving. Which means I have to sit in the car all day. Because I get up very early in the morning, I just want to go to bed and sleep after dinner; I don’t have time to exercise.”* P13

Underestimation of disease severity as a barrier to intention formation: some participants had never contemplated weight loss because experienced no physical discomfort from OSAHS. This underestimation of disease severity is a critical, population-specific barrier. Unlike conditions with overt symptoms, OSAHS can be asymptomatic from the patient’s perspective, especially regarding its long-term cardiovascular risks. This “symptom silence” severs the intuitive link between condition and behavior, meaning that intention formation is not simply weak but actively obstructed by the absence of experiential cues. Interventions must therefore translate invisible risks into personally meaningful experiences, perhaps through personalized health data visualization.

“Well, in fact, I don’t think snoring negatively impacts me, and I don’t feel any uncomfortable. I don’t think it’s a serious disease, so I don’t intend to lose weight.” P18

#### 3.3.4 Reinforcement

Intercurrent health problems as negative reinforcers: a few participants described how new health conditions during weight loss efforts served as powerful disruptors. Conditions such as lumbar disk herniation or ankle injuries effectively punished exercise behavior and led to goal abandonment. This illuminates the precarious nature of weight maintenance in a population vulnerable to multiple comorbidities. Weight loss interventions for this group must be flexible and resilient, incorporating contingency planning for inevitable life disruptions.

*“I was checked out that I had a lumbar disk herniation, which interrupted my exercise program and increased my weight.”* P10

#### 3.3.5 Memory, attention and decision processes

Forgetting and the failure of self-monitoring: participants described intermittent attention to weight loss goals, where initial intentions were gradually eroded by daily life demands. Importantly, they did not describe actively deciding to abandon efforts. Rather, goals simply faded from awareness. This passive drift from behavioral targets suggests interventions cannot rely solely on initial motivation but must embed mechanisms for sustained self-monitoring and environmental cueing. The absence of compensatory strategies further indicates deficits in self-regulatory skills, reinforcing the need for training in these areas.

*“I might remember it(weight loss), but I may forget it after a few days, mainly because I can’t stand the temptation of food.”* P5

#### 3.3.6 Environmental context and resources

Pervasive environmental disruption of weight loss efforts: The most frequently cited barriers related to physical and social environment. Participants described obstacles including work commitments, family obligations, social gatherings, and weather patterns that derailed their plans. What is striking is not merely presence of these barriers but the helplessness they evoked. Participants described disruptions as external forces beyond control, against which personal intentions were powerless. This pattern reflects an external locus of control, where individuals perceive little agency over outcomes. Interventions focusing solely on individual motivation or knowledge are unlikely to succeed without also addressing this learned helplessness and equipping patients with practical strategies to anticipate and navigate unpredictable environments.

*“For example, when there are parties at home during the festival, it’s not easy for me to eat healthy food or control my diet. In this case, I can’t follow my plan to lose weight.”* P25

*“I’m busy at work and lack time to exercise. If I control my diet, I feel hungry and painful, affecting my work efficiency, so I can’t lose weight.”* P13

#### 3.3.7 Social influences

Ambivalent or counterproductive social support: While social support is often framed as uniformly positive, participants’ experiences revealed a more complex picture. Family members, despite good intentions, sometimes unintentionally undermined weight loss efforts through behaviors perceived as caring, such as preparing large meals and encouraging eating. This created tension between health goals and social/family roles. In some cases, family skepticism about the need for weight loss directly contradicted medical advice, placing participants in a bind.

This pattern reveals ambivalent social support–where actions intended as caring (e.g., providing large meals) directly conflict with health goals. This creates a tension between the TDF domain of Social Influences and the domain of Intentions, placing the patient in a bind between social harmony and personal health adherence. It underscores that interventions must extend beyond the individual to engage and educate the social network, aligning their beliefs and behaviors with treatment goals.

*“But as long as I start to lose weight, my father will make a lot of delicious food at home every day, and my husband always let me eat more because they think I’m tired of working, and there is no need to lose weight.”* P8

#### 3.3.8 Emotions

Emotional states as barriers to adherence: participants described bidirectional relationships between emotion and behavior. Negative affective states including fatigue and low mood were reported as triggers for abandoning weight loss efforts, with eating serving as a readily available coping mechanism. This pattern aligns with emotional eating theory, where negative affect prompts consumption as a form of self-regulation. It suggests that for some individuals, weight loss interventions must include components that address emotion regulation directly, rather than assuming that willpower alone can override affective drives.

*“When I feel tired, I don’t want to lose weight and might eat more.”* P4

Weight loss distress and burn-out: additionally, dietary restriction itself was described as emotionally distressing, leading to burn-out. This highlights a critical tension that behaviors required for weight loss may generate negative emotions that undermine adherence. Interventions need to help patients anticipate and manage this emotional toll, framing distress as a normal part of the process rather than a sign of failure.

*“I just feel that it’s too difficult for me. I can’t stand not being allowed to eat.”* P9

Emotional detachment as a protective mechanism: a subset reported emotional detachment from weight loss outcomes, stating they would not be affected by failure. This apparent indifference may represent a protective mechanism against distress from repeated unsuccessful attempts. However, it also signals profound disengagement that may require therapeutic attention before behavior change efforts can be meaningfully initiated.

*“(If the weight loss is not successful) I will not have any feelings.”* P15

### 3.4 Facilitators

#### 3.4.1 Knowledge

Accurate knowledge as a foundation for motivation: in contrast to knowledge deficits described above, participants well-informed about OSAHS and its relationship to obesity demonstrated stronger motivation. Crucially, knowledge alone was not sufficient but appeared necessary. Participants who understood potential consequences of untreated OSAHS, including life-threatening events like sudden death, described this awareness as a powerful driver. Notably, when knowledge was grounded in personal experience, such as noticing snoring worsened with weight gain, it appeared particularly powerful. This suggests educational interventions may be most effective when they make personal health consequences salient and concrete, beyond general information.

*“I know that if snoring is too serious, there will be a lack of oxygen, and long-term respiratory arrest may lead to sudden death.”* P4

“I think they (OSAHS and obesity) might have a certain relationship. Although I used to snore when I was thin, now I feel snoring is serious when I’m fat because once I was suffocated and woke up, I feared dying.” P10

#### 3.4.2 Beliefs about consequences

Positive outcome expectancies driving behavior: participants who believed weight loss would lead to tangible health improvements were more committed to their goals. These positive expectancies were often grounded in personal experience, making them particularly powerful.

*“(If I lose weight) the snoring sound may be lowered because I used to snore more seriously when I was fatter.”* P12

*“(Weight loss) It’s good for my health, such as lowering blood sugar and blood pressure. My physical examination found that my blood sugar is high and related to obesity, so I need to lose weight.”* P4

Dual motivational pathways: additionally, some described anticipated negative consequences of not losing weight as motivating factors. This suggests interventions might leverage both approach-oriented motivations (seeking health gains) and avoidance-oriented motivations (preventing health deterioration).

*“It (obesity) could lead to medical problems, such as heart disease. And it feels hard just to walk long and climb stairs. snoring influences my wife’s sleep.”* P5

Multifaceted nature of outcome expectations: notably, a few mentioned aesthetic and psychosocial benefits, including improved appearance, enhanced self-confidence, and greater daily convenience. This indicates weight loss motivations extend beyond health concerns, and interventions may benefit from acknowledging these personally relevant outcomes.

“(Weight loss) can make me beautiful, my skin and figure will be better, and it’s more convenient to buy clothes.” P8

#### 3.4.3 Social/professional role and identity

Self-regulation as a core identity commitment: almost all participants emphasized that successful weight loss ultimately depended on personal responsibility and self-discipline. This belief in primacy of self-control reflects strong internal locus of control, generally associated with better health outcomes. However, it carries potential risk. If weight loss efforts fail despite strong self-regulation, individuals may internalize this as personal moral failure rather than recognizing environmental and biological influences. Interventions should strengthen this sense of agency while providing realistic perspectives on obesity’s multifactorial nature.

*“I think the most important thing to lose weight is to persevere, you know, I just didn’t lose weight continuously and regularly; that’s why my weight remained high.”* P16

#### 3.4.4 Intentions

Strong, health-anchored intentions: participants with clear weight loss intentions consistently linked goals to health outcomes for themselves and their families. Mention of family members is noteworthy, suggesting prosocial motivations may be particularly powerful. Interventions might explore ways to make these relational benefits more salient, helping patients connect weight loss efforts to valued roles and relationships.

*“Weight loss is mainly for my health, keeping my snoring voice down and letting my wife sleep well.”* P5

#### 3.4.5 Reinforcement

External reinforcement through social support: a few expressed that help and coaching from family and healthcare professionals were important motivations for continuous weight loss. This finding complements the barrier of insufficient social support and underscores potential of structured support programs providing ongoing encouragement and accountability.

*“You know, I think the most important motivation for me is that you have some kind of help and supervision from anyone.”* P1

Internal reinforcement through habit formation: developing a habit was deemed an important prerequisite for weight loss. Automaticity, where behaviors become habitual, was identified as key to sustaining weight loss. This suggests interventions should aim not only to initiate behavior change but to support transition from conscious effort to automatic routine.

“Well, now I exercise daily; morning running has become my habit.” P12

#### 3.4.6 Beliefs about capabilities/optimism

Self-efficacy derived from past successes: participants expressed confidence in their ability to lose weight, often drawing on past successful behavior change in other domains such as smoking cessation. This generalized self-efficacy is an important psychological resource. Interventions might build confidence by helping patients recognize and leverage existing strengths and past successes, even in different behavioral domains. However, confidence must be carefully calibrated. Overconfidence without adequate skills could lead to disappointment and abandonment when challenges arise.

*“Well, I quit smoking successfully, so I think it’s easy for me to lose weight.”* P3

#### 3.4.7 Social influences

Desire for supportive accountability: participants expressed clear preference for support including both encouragement and structure. They specifically mentioned reminders from family and advice from professionals. The concept of “reminders” is significant, implying a need for external support to scaffold the Memory, Attention and Decision Processes identified as barriers.

The expressed need for “reminders” is theoretically significant. It illustrates how a facilitator in the Social Influences domain can directly address a barrier identified in the Memory, Attention and Decision Processes domain (i.e., “forgetting”). This cross-domain interaction highlights the utility of the TDF in designing multifaceted interventions, where external social cues can scaffold self-regulatory deficits that might otherwise undermine sustained behavior change.

*“I think I need the oral remainders from my family members and the professionals’ advice. I think it would be very helpful.”* P5

#### 3.4.8 Emotions

Health-related anxiety as a motivator: many described fears and worry about health consequences of obesity as powerful drivers of weight loss efforts. This finding presents a paradox that can be resolved by attending to temporal focus. Immediate negative affect, such as fatigue or distress, tends to inhibit behavior. In contrast, anticipated negative affect, such as worry about future health outcomes, can serve as a motivational catalyst. Interventions could leverage this distinction by helping patients maintain a vivid connection to long-term health goals while preventing this concern from escalating into paralyzing anxiety.

*“I just worry about my health conditions if I can’t lose weight successfully.”* P21

#### 3.4.9 Goals

Specific goals with flexible implementation: almost all participants had a specific numerical weight target, indicating clear goal intentions. However, they did not rigidly adhere to detailed implementation plans. Instead, they described a more flexible approach of targeting problematic behaviors first. This suggests that while specific challenging goals are motivating, pathways to achieving them may need adaptability. Interventions might encourage clear distal goals while supporting development of flexible personalized proximal plans that accommodate life’s unpredictability.

*“It’s great for me to lose 25 kilos. But I want to eliminate the habit of eating midnight snacks first.”* P8

## 4 Discussion

This study represents the first application of the Theoretical Domains Framework (TDF) to systematically investigate weight-loss behaviors in individuals living with obesity and OSAHS, identifying key determinants across cognitive, behavioral, and environmental dimensions. Through in-depth interpretive analysis grounded in a critical realist epistemology, we identified not only the presence of barriers and facilitators but also the underlying mechanisms through which they operate in this specific clinical population. These findings extend beyond generic subthemes to reveal context-specific insights into weight loss determinants in OSAHS.

This study employed the TDF as the primary analytical lens to systematically identify and categorize determinants. To synthesize these findings into a parsimonious framework for intervention design, we then map them onto the COM-B model’s three essential conditions: Capability, Opportunity, and Motivation. This integration does not alter our findings but provides a higher-order structure to discuss implications. Specifically, knowledge and skills deficits pertain to Psychological Capability; environmental and social barriers relate to Physical and Social Opportunity; and intentions, beliefs, and emotions are core components of Motivation. This dual-framework approach ensures a comprehensive yet organized understanding of intervention targets.

At the cognitive level, prevalent misconceptions among patients (e.g., “surgery is the only solution,” P17) may originate from multiple factors. Our analysis revealed that these knowledge deficits are not merely passive gaps but actively shape treatment preferences in ways that conflict with evidence-based recommendations. Crucially, we found that a knowledge deficit, in the absence of accurate information, can solidify into an active misconception that directly competes with behavioral weight management. This mechanism explains why simply providing general information may be insufficient. This finding extends prior work by highlighting the need for targeted educational interventions addressing specific misconceptions rather than providing general information. These misconceptions likely stem from the insidious nature of OSAHS symptoms leading to underestimation of disease severity, consistent with findings on symptom underestimation (32). Additionally, as previous studies noted, clinicians often prioritize symptomatic management over etiological explanation (33, 34), coupled with insufficient public education regarding the OSAHS-obesity link (3, 35). Crucially, our data suggest that accurate knowledge, while necessary, is insufficient alone. Participants who understood the link between OSAHS and obesity demonstrated stronger motivation, particularly when this knowledge was grounded in personal experience such as noticing that snoring worsened with weight gain. This observation suggests that educational interventions may be most effective when they connect abstract medical knowledge to patients’ lived experiences. Consequently, etiology-based education should be integrated into standard OSAHS care pathways to address these knowledge gaps (36).

Beyond cognitive factors, behavioral challenges highlight structural deficiencies in current weight management services. Our participants’ descriptions of unsuccessful weight loss attempts revealed a critical distinction. These individuals lacked not motivation but the behavioral repertoire necessary for successful sustained weight management. This distinction is conceptually important: it shifts the focus from enhancing motivation to building practical skills and strategies. Their efforts were characterized by *ad hoc* restrictive approaches that proved unsustainable, leading to cycles of weight loss followed by regain accompanied by psychological distress. A meta-analysis by Carneiro-Barrera et al. (15) confirmed that existing weight loss guidance remains overly generalized, lacking OSAHS-specific protocols. Furthermore, research has indicated that the absence of dedicated multidisciplinary OSAHS management teams leaves patients without sustained professional support (37, 38). Our finding that participants explicitly desired systematic professional guidance underscores the urgency of redesigning intervention approaches to include skill-building components that address not only what to do but how to do it sustainably. These insights underscore the imperative to redesign intervention approaches by fostering multidisciplinary collaboration that integrates expertise from pulmonology, nutrition, and psychology. This direction aligns with the emphasis on specialized management in the ACTION-IO study (39).

Environmental barriers emphasize the critical role of support systems. Workplace stress (P13) and inadequate family support (P8) not only directly impede behavior implementation but may indirectly undermine motivation through emotional impacts. Notably, our analysis revealed a more complex picture of social influence than previously reported. Family members despite good intentions sometimes actively undermined weight loss efforts through behaviors perceived as caring such as preparing large meals. This created tension between health goals and social roles, operating at the intersection of the Social Influences and Intentions domains. Participants were placed in a bind between maintaining social harmony and adhering to health behaviors. These findings are corroborated by evidence from prior research investigating workplace-related barriers to weight management (40, 41) and underscoring the critical role of social support in weight maintenance (42). This convergence of evidence underscores the relevance and validates the application of the socioecological model in the context of OSAHS management, thereby highlighting the necessity for establishing and advocating multilevel support networks that actively encompass families, employers, and healthcare institutions.

The observed “intention-behavior gap” reveals limitations of motivational theories. Although outcome expectations were identified as the strongest facilitator (43), cognitive changes alone proved insufficient for sustained behavior change. Our data illuminated this gap through participants’ descriptions of passive drift away from goals, where intentions faded from awareness without conscious decision to abandon efforts. This pattern indicates deficits in self-regulatory skills and sustained self-monitoring, extending beyond simple motivation. It suggests that interventions must embed mechanisms for environmental cueing and sustained self-monitoring, not merely strengthen initial motivation. This result aligns with Chew et al.’s (44) research on motivation stability. This observation not only aligns with but also substantiates self-determination theory, as it suggests that for interventions to be effective, they must be designed to simultaneously foster the core psychological needs of autonomy, competence, and relatedness, a principle that is centrally emphasized in Michie et al.’s (45) taxonomy of behavior change techniques.

Collectively, these findings support the development of a multilevel strategy to advance weight management in OSAHS. Theoretically, integrating the TDF with the COM-B model provides a comprehensive framework for understanding and addressing OSAHS-specific behavioral determinants, offering a structure for applying established implementation principles (22, 46). The depth of our qualitative insights, revealing not just what barriers and facilitators exist but how they operate and interact, provides a foundation for designing theory-based interventions that target specific mechanisms. In clinical practice, this understanding can be operationalized through stepped-care interventions. These interventions logically progress from initial cognitive restructuring and skill-building training to sustained environmental modifications, consistent with phased intervention principles (43). Finally, to ensure widespread and equitable implementation, OSAHS weight management should be formally recognized as an essential component of public health policy and systematically integrated into standard care packages.

This study applied the TDF to examine weight-loss behaviors in individuals living with obesity and OSAHS, enabling systematic identification of barriers and facilitators across cognitive, behavioral, and environmental dimensions. The abductive theoretically-grounded thematic analysis enhanced our understanding of the mechanisms underlying behavior change in this population and provided a foundation for developing theory-based and evidence-informed weight management interventions. Notwithstanding its clear strengths, this study is subject to several potential limitations that warrant consideration. First, recruitment from a single otolaryngology department in one Chinese tertiary hospital may limit generalizability to other geographic regions or healthcare settings. Second, the predominantly male sample (73.1%) and high proportion of severe OSAHS cases may limit transferability to female populations and individuals with mild or moderate OSAHS. Third, as with all qualitative research, the findings are interpretive and context-bound, offering transferable insights rather than statistical generalizability. Fourth, the cross-sectional design captures perspectives at a single time point and cannot track how barriers and facilitators evolve over time; future longitudinal research could illuminate these dynamic processes.

Building on these considerations, future research should pursue several directions. Studies with more diverse samples, including balanced gender representation and varied OSAHS severity, would enhance understanding of how these determinants operate across subgroups. Investigation of female-specific factors (24) and cross-cultural adaptation of interventions (47) are warranted given the gender imbalance in the current sample. The role of digital health technologies such as mHealth in supporting sustained behavior change and providing external reminders for memory and attention processes merits investigation. Additionally, the deeper interpretive themes identified in this analysis, such as the paradoxical role of emotions, the tension between social support and social pressure, and passive goal abandonment, warrant further investigation in larger more diverse samples to examine their prevalence and moderating factors.

## 5 Conclusion

This study demonstrates that weight loss in individuals living with obesity and OSAHS is influenced by multiple interacting factors encompassing capability, opportunity, and motivation dimensions. Through rigorous application of the TDF within an abductive thematic analysis framework, we systematically identified key barriers and facilitators ranging from knowledge misconceptions and skill deficits to environmental disruptions and complex social dynamics. These findings provide preliminary evidence for integrating behavior change techniques into OSAHS weight management strategies. However, given the exploratory nature of this qualitative study and the contextual limitations of the sample, these conclusions should be considered hypothesis-generating rather than definitive. Future research is needed to test the effectiveness of interventions developed based on these insights and examine their applicability across diverse populations and settings.

## Data Availability

The original contributions presented in this study are included in this article/Supplementary material, further inquiries can be directed to the corresponding authors.
