# Supplementary material for: Barriers and facilitators to weight loss in patients living with obesity and OSAHS: a qualitative study based on the Theoretical Domains Framework (TDF)
Source: Front Med (Lausanne). 2026 Apr 24;13:1782833. doi: 10.3389/fmed.2026.1782833 (PMC13152744; doi:10.3389/fmed.2026.1782833)
Supplement: Supplementary file 1 [file Table_1.docx]

Supplementary Material

# Supplemental Appendix S1：Semi structured interview questions

**Knowledge/Beliefs about Consequences**

1. What do you know about OSAHS? Do you consider your weight to be normal, overweight or obese?

2. Do you think there is a relationship between obesity and OSAHS? Do you think that weight loss is beneficial to improve OSAHS? What are the specific benefits? What do you think affects your health if you do not lose weight?

3.What do you know about weight loss methods?

**Skills/Behavioural Regulation**

4. If you have tried to lose weight, have you received professional guidelines for losing weight? What are the specific guidelines? Did you follow them?

5. Do you have the necessary skills/techniques to help you lose weight? What professional guidelines do you think you need for losing weight?

**Social/Professional Role and Identify**

6. Could you tell me what is your responsibility during the process if you decide to lose weight?

**Intentions/Reinforcement**

7. For what specific incentives or disincentives do you decide to start losing weight?

8. Could anything help you lose weight in the long term?

**Beliefs about Capabilities/Optimism**

9. How confident do you feel in losing weight? Can you think of anything that could help you become more confident?

10. What influences how self-confident you feel?

**Memory, Attention and Decision Processes**

11. Do you think you will remember to follow the weight loss program? Can you name reasons for why you forget to follow it?

12. What might be the most effective way to follow the weight loss plan?

**Environmental Context and Resources**

13. What are the barriers/facilitators of your life/work environment to losing weight?

14. What resources help in the execution of losing weight? (e.g., professional guidelines, social support, supervision)

**Social Influences**

15. Did others stress the importance of losing weight to you? If you have tried to lose weight, how did they help you?

16. What help would you need to lose weight from others?

**Emotions**

17. If you have tried to lose weight, how did you feel during the process?

18. Is there anything you are afraid of in losing weight?

**Goals**

19. What goals/plans do you have regarding weight loss?

20. That is all my questions. Is there anything else I should know about your weight loss experience?

**Supplemental Appendix S2: Consolidated Criteria for Reporting Qualitative Research (COREQ) checklist**

A checklist of items that should be included in reports of qualitative research. You must report the page number in your manuscript where you consider each item listed in this checklist. If you have not included this information, revise your manuscript accordingly before submitting or note N/A.

| **Topic** | **Item No.** | **Guide Questions/Description** | **Reported on Page No.** |
| --- | --- | --- | --- |
| **Domain 1: Research team and reflexivity** | | | |
| *Personal characteristics* | | | |
| Interviewer/Facilitator | 1 | Which author/s conducted the interview or focus group? | 3-4 |
| Credentials | 2 | What were the researcher’s credentials? E.g. PhD, MD | 3-4 |
| Occupation | 3 | What was their occupation at the time of the study? | 3-4 |
| Gender | 4 | Was the researcher male or female? | N/A |
| Experience and training | 5 | What experience or training did the researcher have? | 3-4 |
| *Relationship with participants* | | | |
| Relationship established | 6 | Was a relationship established prior tostudy commencement? | 3-4 |
| Participant knowledge of the interviewer | 7 | What did the participants know about the researcher? e.g. personal goals, reasons for doing the research | 3-4 |
| Interviewer characteristics | 8 | What characteristics were reported about the interviewer/facilitator? e.g. Bias, assumptions, reasons and interests in the research topic | 3-4 |
| **Domain 2: Study design** | | | |
| *Theoretical framework* | | | |
| Methodological orientation and Theory | 9 | What methodological orientation was stated to underpin the study? e.g. grounded theory, discourse analysis, ethnography, phenomenology, content analysis | 2-4 |
| *Participant selection* | | | |
| Sampling | 10 | How were participants selected? e.g. purposive, convenience, consecutive, snowball | 3 |
| Method of approach | 11 | How were participants approached? e.g. face-to-face, telephone, mail, email | 3 |
| Sample size | 12 | How many participants were in the study? | 4 |
| Non-participation | 13 | How many people refused to participate or dropped out? Reasons? | 4 |
| *Setting* | | | |
| Setting of data collection | 14 | Where was the data collected? e.g. home, clinic, workplace | 3-4 |
| Presence of non-participants | 15 | Was anyone else present besides the participants and researchers? | 3-4 |
| Description of sample | 16 | What are the important characteristics of the sample? e.g. demographic data, date | 4 |
| *Data collection* | | | |
| Interview guide | 17 | Were questions, prompts, guides provided by the authors? Was it pilot tested? | 3 |
| Repeat interviews | 18 | Were repeat interviews carried out? If yes, how many? | 4 |
| Audio/visual recording | 19 | Did the research use audio or visual recording to collect the data? | 3 |
| Field notes | 20 | Were field notes made during and/or after the interview or focus group? | 3-4 |
| Duration | 21 | What was the duration of the interviews or focus group? | 4 |
| Data saturation | 22 | Was data saturation discussed? | 3-4 |
| Transcripts returned | 23 | Were transcripts returned to participants for comment and/or correction? | 3-4 |
| **Domain 3: Analysis and findings** | | | |
| *Data analysis* | | | |
| Number of data coders | 24 | How many data coders coded the data? | 4 |
| Description of the coding tree | 25 | Did authors provide a description of the coding tree? | 4-5 |
| Derivation of themes | 26 | Were themes identified in advance or derived from the data? | 5-8 |
| Software | 27 | What software, if applicable, was used to manage the data? | N/A |
| Participant checking | 28 | Did participants provide feedback on the findings? | N/A |
| *Reporting* | | | |
| Quotations presented | 29 | Were participant quotations presented to illustrate the themes/findings? Was each quotation identified? e.g. participant number | 5-8 |
| Data and findings consistent | 30 | Was there consistency between the data presented and the findings? | 8-9 |
| Clarity of major themes | 31 | Were major themes clearly presented in the findings? | 4-8 |
| Clarity of minor themes | 32 | Is there a description of diverse cases or discussion of minor themes? | 4-8 |

Developed from: Allison Tong, Peter Sainsbury, Jonathan Craig, Consolidated criteria for reporting qualitative research (COREQ): a 32-item checklist for interviews and focus groups, International Journal for Quality in Health Care, Volume 19, Issue 6, December 2007, Pages 349–357, https://doi.org/10.1093/intqhc/mzm042
